# Granulomas Galore: Concomitant Granulomatous Infections in a Patient With Crohn's Disease

**DOI:** 10.7759/cureus.54225

**Published:** 2024-02-15

**Authors:** Michael Gianarakis, Alexander Gianarakis, Safia Ahmed, John Pueringer, Ushan Ranasinghe

**Affiliations:** 1 Internal Medicine, University of Illinois at Chicago, Chicago, USA; 2 Family Medicine, St. George's University of London, London, GBR; 3 Family Medicine, Swedish Hospital, Chicago, USA; 4 Internal Medicine, Swedish Hospital, Chicago, USA

**Keywords:** anti-fungal therapy, anti-tuberculosis therapy, adalimumab (humira), histoplasmosis, tuberculosis, crohn’s disease (cd), tumor necrosis factor-alpha (tnf-α) inhibitors

## Abstract

Tumor necrosis factor (TNF)-alpha inhibitors are effective biologics in the treatment of inflammatory bowel disease; however, they increase susceptibility to opportunistic infections. We report a case of a 74-year-old female with Crohn's disease who developed concomitant pulmonary tuberculosis (*Mycobacterium tuberculosis* [MTB]) and *Histoplasmosis capsulatum* infection while on adalimumab. Co-infection is rare in patients on TNF-alpha inhibitor therapy, and most cases have been reported in patients with human immunodeficiency virus (HIV). This was a challenging case for diagnosis and treatment due to indistinguishable presenting symptoms of both infections, similar laboratory and radiographical findings, and a clinical course complicated by drug-drug interactions and worsening of symptoms despite therapy.

## Introduction

Tumor necrosis factor (TNF)-alpha inhibitors have improved the therapeutic outcomes in inflammatory bowel disease (IBD) treatment; however, they have been associated with severe adverse effects, including malignant neoplasms and opportunistic infections, such as *Mycobacterium tuberculosis* (MTB) and *Histoplasmosis capsulatum*. TNF-alpha is an important cytokine that promotes and maintains granuloma formation and is a critical component of host immunity against pathogens such as mycobacteria and fungi [1]. MTB and histoplasmosis co-infection in patients with human immunodeficiency virus (HIV) has been well-documented in the literature; however, such co-infections are rare in patients treated with TNF inhibitors.

This case report was previously presented as a meeting abstract at the 2022 American College of Gastroenterology Annual Meeting on October 24, 2022.

## Case presentation

A 74-year-old female with a nine-year history of diffuse Crohn's disease of the small and large intestine presented to the emergency department after being referred by her primary care physician for a hemoglobin (Hb) level of 6 g/dL on outpatient laboratory testing.

She reported progressively worsening generalized weakness, headache, dyspnea on exertion, dry cough, fatigue, and a 30 lb weight loss in the three months before presentation. Additionally, she reported the onset of subjective fevers, chills, and dizziness in the three days preceding the presentation. The patient denied any episodes of hematochezia, melena, or hemoptysis.

The patient failed mesalamine treatment for Crohn's disease and was currently being treated with adalimumab (40 mg subcutaneous injection every two weeks), which was commenced nine months before presentation. Interferon-gamma release assay (IGRA), Hepatitis B surface antigen (HBsAg), anti-Hepatitis C virus (HCV), and HIV testing were negative before commencing adalimumab. The patient self-discontinued adalimumab after six months due to fatigue and diarrhea. She presented to her gastroenterologist and was found to have an elevated fecal calprotectin to 522 µg/g. She was treated for a Crohn's disease flare with a corticosteroid taper. Her symptoms improved, and adalimumab was resumed two weeks before the presentation.

**Figure 1:**
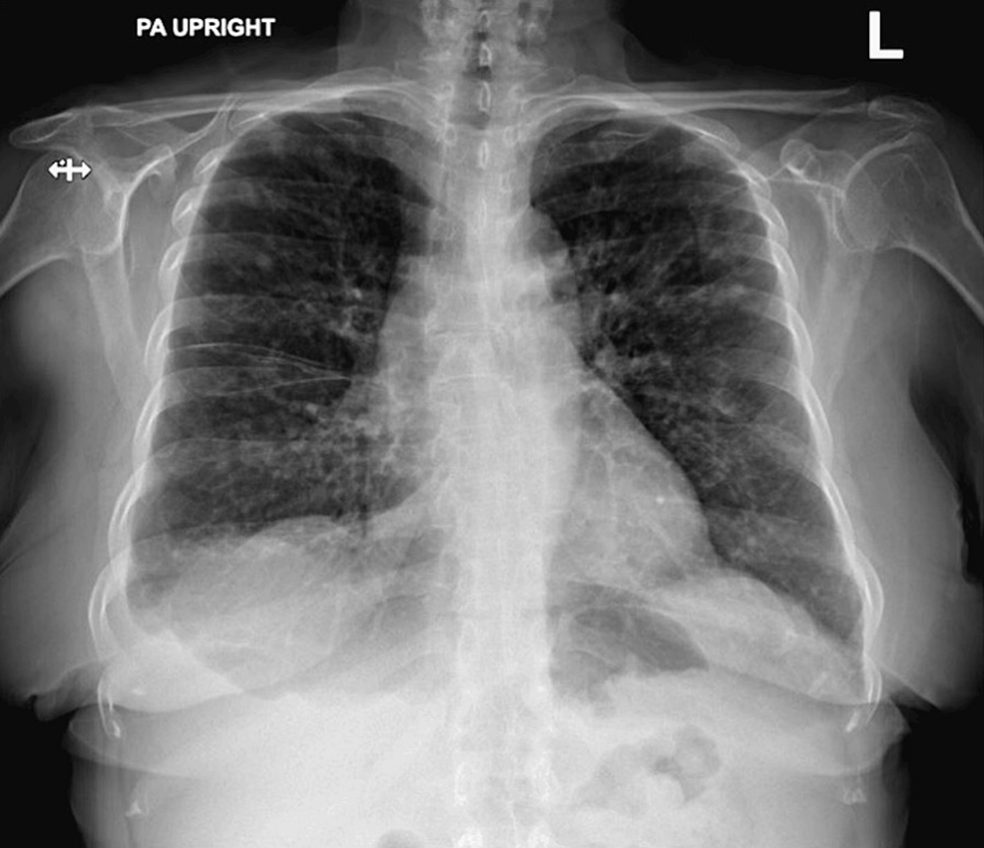
Chest radiography showing a small right pleural effusion and bilateral reticular pulmonary opacities.

Laboratory investigations on presentation showed the following results: white blood cell count (WBC) 5.26 x 10^3 ^µL^-1^, Hb 6.0 g/dL, mean corpuscular volume (MCV) 72 fL, aspartate aminotransferase (AST) 89 U/L, alanine aminotransferase (ALT) 83 U/L, alkaline phosphatase (ALP) 666 U/L, C-reactive protein (CRP) 130.7 mg/L, and lactate dehydrogenase (LDH) 230 U/L. Physical examination was remarkable for decreased breath sounds in the bilateral lung and lower lung fields. Chest radiograph showed a small right pleural effusion and diffuse, bilateral reticular pulmonary opacities (Figure 1). She received one unit of packed red blood cells (pRBCs) and was hospitalized for treatment of anemia, evaluation of elevated liver enzymes, and pleural effusion.

**Figure 2:**
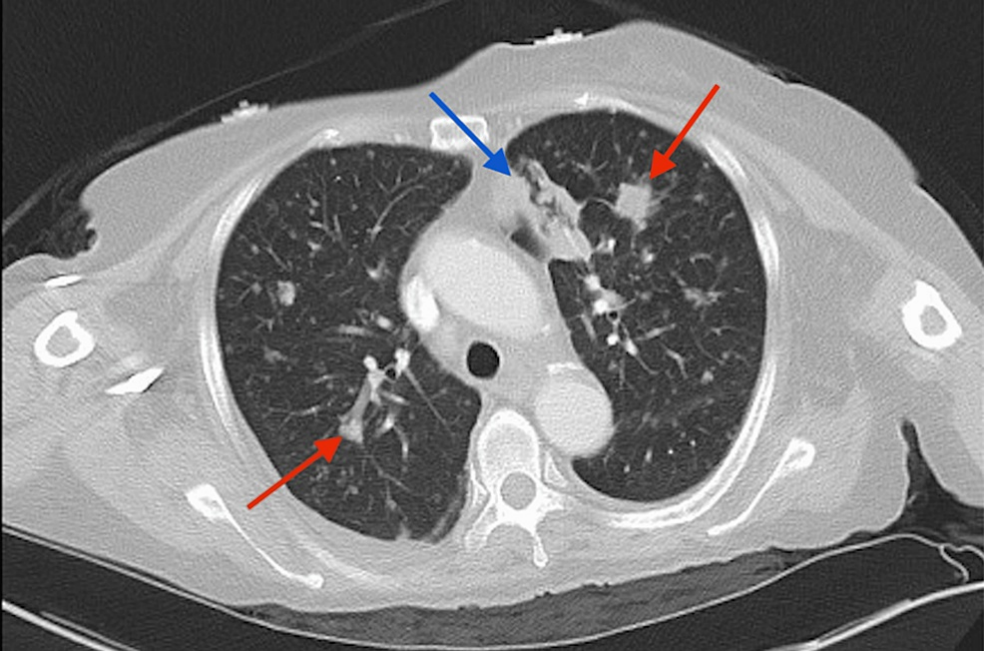
Computed tomography of the chest on day 2 of admission demonstrating bilateral spiculated ground glass pulmonary opacities (red arrows), pleural thickening of the left upper lobe pericardiac region (blue arrows), and mediastinal lymphadenopathy.

Abdominal computed tomography (CT) showed irregular bowel wall thickening in the large and small bowel most prominent in the cecum and distal small bowel with no evidence of abscess or bowel obstruction. Chest CT revealed bilateral ground glass spiculated pulmonary nodules, mediastinal lymphadenopathy, and pleural thickening in the left upper lobe (Figure 2). Adalimumab was held in the setting of suspected infection versus malignancy.

A colonoscopy was performed and showed scattered ulcerations throughout the colon, consistent with prior colonoscopies. There was no evidence of gastrointestinal bleeding.

Bronchoscopy with bronchoalveolar lavage (BAL) of the right upper lobe anterior subsegment and endobronchial ultrasound with transtracheal and transbronchial biopsy were performed. BAL gram stain showed less than 25 WBC per low-power field (LPF) and no evidence of bacteria. Acid-fast bacilli (AFB) smear and initial fungal smears were negative. On day 3 of admission, she began to develop a high-grade fever (102.9 °F), and empiric antibiotics (intravenous vancomycin 750 mg daily, intravenous ceftazidime 1 mg twice daily, and intravenous doxycycline 100 mg twice daily) were commenced. On day 4 of admission, the patient developed a dry cough and SpO2 of 88% on room air. The patient was placed on oxygen therapy with a 4-L nasal cannula, and intravenous liposomal amphotericin B at 3 mg/kg daily was commenced due to a high clinical suspicion of fungal infection. Renal function and electrolytes were closely monitored throughout intravenous liposomal amphotericin B treatment. 

**Figure 3:**
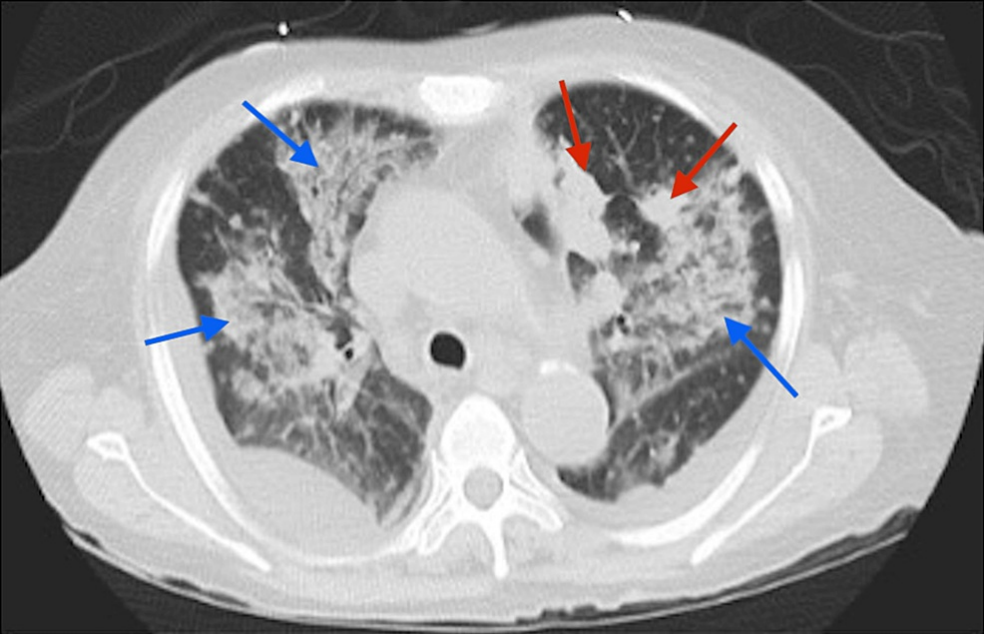
Computed tomography of the chest on day 7 of admission identifying prior nodular opacities (red arrows) and new confluent areas of ground glass opacities with intervening interstitial thickening (blue arrows).

On day 6 of admission, the methenamine silver stain of bronchial washings revealed small budding yeast forms consistent with histoplasmosis. The 1-3-beta-D-glucan assay yielded a positive result (>80 pg/mL). The urine histoplasmosis antigen was negative. Cytology of BAL fluid and transbronchial tissue biopsies returned negative for malignancy. On day 7 of admission, repeat CT imaging revealed prior nodular opacities and worsening confluent areas of ground glass nodular opacities in the bilateral lung fields, with intervening areas of interstitial thickening (Figure 3).

The PaO2 to FiO2 ratio was 160, and corticosteroid therapy was initiated due to concerns for early acute respiratory distress syndrome (ARDS). Secondary infection was suspected, and a rapid MTB polymerase chain reaction (PCR) assay from both the bronchial washings of the right upper lobe lesion and transbronchial tissue returned positive, confirming MTB co-infection. The patient commenced on the following oral drug regimen: rifampin 600 mg daily, isoniazid 300 mg daily, pyridoxine 50 mg daily, pyrazinamide 1,000 mg daily, and ethambutol 800 mg daily.

The patient's symptoms began to improve eight to nine days after the initiation of treatment and supplemental oxygen was weaned off. After 13 days of liposomal amphotericin B treatment, histoplasmosis treatment was transitioned to oral itraconazole 200 mg twice daily for 12 weeks. Due to rifampin's interaction with itraconazole, serum itraconazole levels were monitored on discharge. The patient was discharged on day 17.

The patient was readmitted after 10 days with complaints of recurring fever, night sweats, sore throat, nonproductive cough, and generalized weakness. Vital signs were remarkable for temperature of 101.8 °F and heart rate of 123 beats per minute (bpm). The electrocardiogram showed sinus tachycardia. Laboratory investigations were remarkable for Hgb 7.1 g/dL, and the patient was transfused two units of pRBCs. WBC on admission was 8.2 x 10^3 ^µL^-1^. On day 2 of admission, the patient developed increased work of breathing and hypoxia with SpO2 of 85% requiring up to 6 L oxygen via nasal cannula. Fevers persisted with a maximum temperature of 104.4 °F. 

Chest radiograph showed worsening diffuse, bilateral alveolar, and interstitial infiltrates and focal consolidation in the left upper lobe. Serum itraconazole levels were subtherapeutic at <0.1 mg/L, and treatment was switched to intravenous liposomal amphotericin B 3 mg/kg daily. 

On day 3 of admission, the patient's fevers persisted, and WBC increased to 15.92 x 10^3 ^µL^-1^. Intravenous vancomycin 15 mg/kg every 12 hours and cefepime 2 g every eight hours were commenced with concern for superimposed hospital-acquired pneumonia. Immune reconstitution inflammatory syndrome (IRIS) in the setting of TNF-alpha inhibitor discontinuation was also considered. Blood, urine, and sputum cultures were negative. The respiratory viral panel was negative. 

The patient's respiratory status gradually improved, and by day 8 of admission, she was weaned to 2 L of oxygen via nasal cannula, fevers had resolved, and WBC had normalized. The remainder of her hospitalization was complicated by recurrent melena with anemia (Hb 6.1 g/dL), necessitating pRBC transfusion. Esophagogastroduodenoscopy (EGD) revealed a bleeding duodenal bulb ulcer that was successfully treated with electrocautery and endoscopic clipping. 

Following stabilization of Hb and resolution of MTB antibiotic susceptibilities, the patient was discharged. The patient's MTB strain was pan-susceptible, and she was discharged with oral moxifloxacin 400 mg daily substituted for rifampin given its interaction with itraconazole. She followed up with the health department for direct observed therapy.

## Discussion

Concomitant MTB and histoplasmosis have predominantly been reported in patients with HIV, with an estimated 8%-15% of histoplasmosis-infected HIV patients having MTB co-infection [2]. One case of TNF-alpha inhibitor-associated co-infection in a patient with rheumatoid arthritis was reported; however, this patient had multiple positive tuberculin skin tests (TST) before TNF inhibitor commencement and was not treated for latent MTB [3]. Our case is unique as our patient was HIV-negative and had a negative IGRA before therapy. IGRA is a more specific and sensitive test than TST for detecting latent infection, particularly if patients are on immunosuppressive therapy or have had the BCG vaccine [4].

High clinical suspicion for co-infection should be exercised, particularly in complex disease courses. Co-infection presents challenges for diagnosis due to similarities in clinical presentation, including fever, dyspnea, cough, weight loss, night sweats, fatigue, hepatosplenomegaly, and lymphadenopathy [5]. Pancytopenia, increased liver enzymes, and elevated CRP and ESR are notable in both infections [2]. The lung nodules, granulomas, and infiltrates of both histoplasmosis and MTB present similarly on chest radiography and can mimic malignancy [6]. Both MTB and histoplasmosis are slow-growing organisms, which may delay diagnosis. Furthermore, histoplasmosis urine antigen was negative in our case and may only be detected in 40% of acute localized pulmonary diseases due to a lower fungal burden [7].

The interaction between rifampin and itraconazole is another significant challenge of co-infection and was likely a contributing factor to the patient's readmission [8]. Physicians need to observe for signs of therapeutic failure and monitor serum itraconazole concentrations where possible. It is also important to consider alternative therapy in co-infection due to this significant drug-drug interaction. Moxifloxacin is an effective substitute for rifampin, allowing a good response to itraconazole therapy for histoplasmosis [9].

Although likely better explained by treatment failure, IRIS was considered a possible explanation for our patient's respiratory deterioration during her second admission. IRIS was first described after HIV patients with concurrent infections exhibited worsening symptoms after commencing antiretroviral therapy [10]. More recently, IRIS following discontinuation of TNF-alpha inhibitors has been described, with a median onset of six weeks [11]. Paradoxical worsening of symptoms in TNF-alpha inhibitor-associated infections may be interpreted as treatment failure or additional infections; however, it is important to consider IRIS.

Our patient was likely exposed to histoplasmosis in the community due to its endemicity in the Midwest USA. There are currently no guidelines or evidence that support histoplasmosis screening before TNF-alpha inhibitor initiation [7]. An IGRA to detect asymptomatic individuals has been studied; however, further evaluation of its effectiveness is necessary [12]. Patients should be screened before and throughout TNF-alpha inhibitor therapy for symptoms of infection and exposure, concerning high-risk activities, such as traveling to endemic areas, old buildings, animal coops, and caves.

Screening for MTB before TNF-alpha inhibitor initiation is well-established. However, there are currently no evidence-based guidelines and few reports evaluating re-screening for newly acquired MTB infection during therapy [13]. The American College of Rheumatology recommends annual testing in patients on biological therapy who live, work, or travel to MTB endemic areas [14]. Our case indicates that similar recommendations and a re-screening strategy should be supported within the field of gastroenterology. Clinicians should perform annual risk stratification with focused TB exposure history and subsequent testing where necessary. Risk factors for developing MTB with TNF-alpha inhibitor therapy include concomitant use of immunosuppressants, travel to endemic areas, healthcare workers, and a history of active/latent MTB [15]. Further research is required to determine a sensitive and cost-effective re-screening methodology.

## Conclusions

This case recognizes a rare presentation of concomitant granulomatous infections in an HIV-negative patient treated with TNF-alpha inhibitor therapy for Crohn's disease. The diagnosis and treatment of co-infection are challenging due to overlapping symptoms, similar laboratory and imaging findings, and drug-drug interactions. Physicians must exercise high clinical suspicion and evaluate for MTB exposure risk throughout treatment with TNF-alpha inhibitors. Furthermore, co-infection with histoplasmosis should be considered, particularly in endemic regions, and early recognition is imperative, particularly in patients with a complicated or treatment-refractory disease course to ensure prompt initiation of appropriate treatment. In co-infection, alternate therapies should be considered due to itraconazole-rifampin drug interaction. Guidelines recommending annual re-screening for MTB during TNF-alpha inhibitor treatment are necessary for patients with a high susceptibility to exposure, whether through occupation, living or traveling in endemic areas, or contact with exposed individuals.
